# Effect of shallow angles on compressive strength of biaxial and triaxial laminates

**DOI:** 10.1186/s40064-016-3726-8

**Published:** 2016-11-30

**Authors:** Hongli Jia, Hyun-Ik Yang

**Affiliations:** Department of Mechanical Engineering, Hanyang University, Room 224, Engineering Building 5, 55 Hanyangdeahak-ro, Sangnok-gu, Ansan, Gyeonggi-do 15588 Republic of Korea

**Keywords:** Shallow-angled laminate, Compression, Strength, Failure criterion

## Abstract

**Background:**

Biaxial (BX) and triaxial (TX) composite laminates with ±45° angled plies have been widely used in wind turbine blades. As the scale of blades increases, BX and TX laminates with shallow-angled plies (i.e. off-axis ply angle <45°) might be utilized for reducing mass and/or improving performance. The compressive properties of shallow-angled BX and TX laminates are critical considering their locations in a wind turbine blade, and therefore in this study, the uniaxial static compression tests were conducted using BX and TX laminates with angled-plies of ±45°, ±35°, and ±25°, for the purpose of evaluation. On the other hand, Mori–Tanaka mean field homogenization method was employed to predict elastic constants of plies in BX and TX laminates involved in tests; linear regression analyses of experimentally measured ply strengths collected from various sources were then performed to estimate strengths of plies in BX and TX laminates; finally, Tsai–Wu, Hashin, and Puck failure criteria were chosen to predict compressive strengths of BX and TX laminates. Comparison between theoretical predictions and test results were carried out to illustrate the effectiveness of each criterion.

**Results:**

The compressive strength of BX laminate decreases as ply angle increases, and the trend was successfully predicted by all three failure criteria. For TX laminates, ±35° angled plies rather than ±45° angled plies led to the lowest laminate compressive strength. Hashin and Puck criteria gave good predictions at certain ply angles for TX laminates, but Tsai–Wu criterion was able to capture the unexpected strength variation of TX laminates with ply angle.

**Conclusion:**

It was concluded that the transverse tensile stress in 0° plies of TX laminates, which attains its maximum when the off-axis ply angle is 35°, is the dominant factor in failure determination if using Tsai–Wu criterion. This explains the unexpected strength variation of TX laminates with ply angle, and also indicates that proper selection of ply angle is the key to fully utilizing the advantages of shallow-angled laminates.

## Background

Polymeric matrix composites (PMCs), especially the continuous fiber reinforced plastics, feature excellent specific stiffness and strengths along the longitudinal direction. Since a composite ply exhibits distinct properties in the longitudinal and transverse directions, composite laminates consisting of stacked plies with different orientations can be created to satisfy specific requirements on stiffness and strength along certain directions. Biaxial (BX) and triaxial (TX) laminates with stacking sequences of [±*θ*_*m*_]_S_ and [0_*m*_/±*θ*_*n*_]_S_ are two types laminates frequently encountered in various industrial applications, especially when *θ* = 45°. One typical application of TX and BX laminates is wind turbine blades, where TX laminates are used in the skin, and BX laminates are used in the shear web. As the scale of blades increases, it is always preferred to have a lighter yet stronger structure with lower cost. At the same time, accurate estimation of laminate strengths is crucial to achieve the goal.

Azzi and Tsai (1965) proposed a quadratic failure criterion for transversely isotropic materials such as unidirectional composites (UDs) based on Hill’s yield criterion for orthotropic material (Hill 1950), and this criterion was then refined to become Tsai–Hill criterion (Tsai 1965). Tsai and Wu (1971, 1972) re-derived a quadratic failure criterion using a scalar function of two strength tensors, which was later named after the two contributors, and is still one of the most widely employed failure criterion. Comparing with Tsai–Hill criterion, Tsai–Wu criterion incorporates different tensile and compressive strengths, and includes one linear invariant, as well as different terms describing interaction between normal stress components. Other well-known quadratic failure criteria include Hoffman (1967), which differs from Tsai–Wu only in the interaction terms, and Chamis (1969), which is similar to Tsai–Hill but with different interaction terms.

Since composites are microscopically heterogeneous, it would be of practical importance to predict failure modes or failed constituents. Unfortunately, all quadratic failure criteria mentioned above do not possess such capability. The earliest successful endeavor was made by Hashin and Rotem (1973), who proposed four distinct failure modes, i.e. fiber tension, fiber compression, matrix tension, matrix compression, and their respectively associated failure criteria. Note that in-plane shear stress only contributes to matrix failure in those criteria. Hashin (1980) further developed those four criteria, which were expressed in terms of transversely isotropic stress invariants, to cover three-dimensional stress states applied to a UD.

In addition to the aforementioned pioneering work, Puck and Schürmann (1998, 2002) and Puck et al. (2002) proposed a set of failure criteria for UD considering two types of failure: fiber failure (FF) and inter-fiber failure (IFF). Effect of fiber kinking under compression is included in FF, while three different fracture modes are distinguished in IFF depending on the direction of transverse stress as well as the ratio between the magnitudes of the transverse and in-plane shear stresses. Dávila and Camanho (2003) and Dávila et al. (2005) developed a set of six failure criteria referred as LaRC03, corresponding to 3 fiber failure modes and 3 matrix failure modes of UD, respectively. Three matrix failure criteria include one for transverse tension, derived based on Dvorak and Laws’ (1987) analysis of transverse crack propagation in a ply, and two quadratic criteria derived based on Mohr–Coulomb criterion and Puck’s action plane concept (Puck and Schürmann 1998), corresponding to transverse compression accompanied by two different longitudinal stress states. Three fiber failure criteria include one for longitudinal tension, and two for fiber kinking under longitudinal compression with respective matrix tension and compression. LaRC03 was also updated to LaRC04 (Pinho et al. 2005) to cover three-dimensional stresses.

Sun et al. (1996) and París (2001) offered comprehensive overview of existing failure criteria for composites, and the first World-Wide Failure Exercise (WWFE-I) evaluated the predictive capability of a number of failure theories by comparing predictions with experimental results (Soden et al. 1998, 2004; Hinton et al. 2002, 2004; Kaddour et al. 2004).

Recently, Hayat and Ha (2015) and Ha et al. (2014) proposed the utilization of shallow-angled BX and TX laminates in large-scale wind turbine blades in order to reduce weight and/or improve performance. Considering the locations of BX and TX laminates in a wind turbine blade, their performance under compression is critical. Therefore, in this study, their performance under static compressive loading was measured. Afterwards, three ply-based failure criteria (Tsai–Wu, Hashin 2D, Puck) were chosen to predict experimentally measured compressive strengths of BX and TX laminates. Predicted values were compared with test data to illustrate the effectiveness of each criterion. Analysis was performed to unveil the cause of unexpected variation of TX compressive strength with ply angle.

## Theory

### Estimation of elastic constants

To estimate elastic constants of UD from those of constituents, a number of micromechanical methods can be utilized, ranging from rule-of-mixture to finite element models of UD micro-structure. In this work, Mori–Tanaka-type (1973) mean field homogenization method was chosen due to the preference for a closed-form expression with sound physical basis and proven accuracy. The stiffness tensor of a UD composite ${\mathbf{C}}_{\text{comp}}$ with fiber volume fraction $V_{\text{f}}$ is given below (Tandon and Weng 1984):

1 $${\mathbf{C}}_{\text{comp}} = {\mathbf{C}}_{\text{m}} \left\{ {{\mathbf{I}} - V_{\text{f}}\,\left[ {\left( {{\mathbf{C}}_{\text{f}} - {\mathbf{C}}_{\text{m}} } \right)\left( {{\mathbf{S}} - V_{\text{f}} \left( {{\mathbf{S}} - {\mathbf{I}}} \right)} \right) + {\mathbf{C}}_{\text{m}} } \right]^{ - 1} \left( {{\mathbf{C}}_{\text{f}} - {\mathbf{C}}_{\text{m}} } \right)} \right\}^{ - 1}$$

where ${\mathbf{C}}_{\text{f}}$ and ${\mathbf{C}}_{\text{m}}$ are stiffness tensors of the composite, fiber, and matrix, respectively; **I** is the identity tensor; **S** is Eshelby’s tensor, the detailed formulation of which for a cylinder can be found elsewhere (Eshelby 1957; Mura 1987). The elastic constants of UD glass/epoxy composites involved in this study were estimated using the above-mentioned approach, and the corresponding numerical values are listed in Table 2.

### Estimation of ply strengths

In order to predict strengths of multi-directional laminates with ply-based failure criteria, ply strengths are required. Despite the fact that several micromechanical approaches are available, such as equations from Chamis (1984), estimating ply strengths from constituent strengths still remains an issue. Extensive experimental evidences have revealed, and it has been recognized (Kelly 1984) that, given the same fiber and matrix with a reasonable $V_{\text{f}}$ (usually below 0.7), the ply longitudinal tensile and compressive strengths are approximately linear to $V_{\text{f}}$; the transverse tensile and compressive strengths respectively decreases and increases with increasing $V_{\text{f}}$. Therefore, instead of employing complex micromechanical models for ply strength prediction, which is out of the scope of the current work, it was assumed that each ply strength was linearly proportional to $V_{\text{f}}$. Simple linear regression analyses were performed over a collection of UD strength test data, to estimate ply strengths at different $V_{\text{f}}$, which were then used as inputs to ply-based failure criteria to predict strengths of multi-directional laminates. The UD strength test data collected from literature are shown in Table 1, and the ply strengths at various $V_{\text{f}}$ estimated based on those collected test data, are tabulated in Table 2.

**Table 1:** Strengths of UD glass/epoxy with different *V*_f_

*V*_f_	*X*_T_ (MPa)	*X*_C_ (MPa)	*Y*_T_ (MPa)	*Y*_C_ (MPa)	*S* (MPa)
0	68 (Momentive Specialty Chemicals Inc. 2006)	85 (Momentive Specialty Chemicals Inc. 2006)	68 (Momentive Specialty Chemicals Inc. 2006)	85 (Momentive Specialty Chemicals Inc. 2006)	–
0.5	1215 (Rolfes et al. 2008)	–	39.7 (Rolfes et al. 2008)	91 (Rolfes et al. 2008)	51.2 (Rolfes et al. 2008)
0.515	–	–	36.7 (Wichmann 2002)	–	–
0.6	1200 (Owens Corning Composite Materials 2011)	721^a^	32.6 (Rolfes et al. 2008)	105.9 (Rolfes et al. 2008)	52.7 (Rolfes et al. 2008)
0.638	1430 (Peters et al. 2009)	850 (Peters et al. 2009)	–	–	–
1	2430 (Owens Corning Composite Materials 2008)	1640^a^	–	–	62.7 (Owens Corning Composite Materials 2008)
*r*	0.989	0.971	−0.998	0.820	0.998

^a^The value of $X_{\text{C}}$ was estimated using $X_{\text{T}}$ from Owens Corning Composite Materials (2008) and the ratio between $X_{\text{T}}$ and $X_{\text{C}}$ for E-glass fiber from Kaddour and Hinton (2012)

**Table 2:** Predicted ply elastic constants and in-plane strengths for BX and TX laminates

Laminate	*V*_f_	*E*_11_ (GPa)	*E*_22_ (GPa)	*ν*_12_	*G*_12_ (GPa)	*X*_T_ (MPa)	*X*_C_ (MPa)	*Y*_T_ (MPa)	*Y*_C_ (MPa)	*S* (MPa)
BX25	0.49	41.6	8.1	0.27	3.0	1093.4	778.2	40.2	97.4	50.6
BX35	0.44	37.7	7.2	0.27	2.7	985.9	703.8	43.0	96.0	49.4
BX45	0.39	33.9	6.5	0.28	2.4	877.8	628.9	46.0	94.7	48.3
TX25	0.54	45.2	9.1	0.26	3.4	1193.9	847.8	37.5	98.7	51.7
TX35	0.53	44.7	9.0	0.26	3.3	1181.8	839.5	37.8	98.5	51.5
TX45	0.56	46.8	9.6	0.26	3.5	1238.8	878.9	36.2	99.2	52.1

### Failure criteria

In this study, three ply-based failure criteria: Tsai–Wu, Hashin 2D, and Puck, were selected by taking into account their predictive capabilities (Soden et al. 1998, 2004; Hinton et al. 2002, 2004; Kaddour et al. 2004) as well as ease of application, to predict uniaxial compressive strengths of BX and TX laminates.

 The Tsai–Wu (1971, 1972) failure criterion in 2D case is given in Eq. (2), where $\sigma_{1}$, $\sigma_{2}$, and $\sigma_{6}$ represent ply longitudinal, transverse, and in-plane shear stresses, respectively. All coefficients in Eq. (2) are defined in Eq. (3), where $X_{\text{T}}$, $X_{\text{C}}$, $Y_{\text{T}}$, $Y_{\text{C}}$, and *S* are in-plane strengths corresponding to longitudinal tension, longitudinal compression, transverse tension, transverse compression, and in-plane shear, respectively. All strengths take absolute values, while all stresses keep their signs.

2 $$F_{11} \sigma_{1}^{2} + F_{22} \sigma_{2}^{2} + F_{66} \sigma_{6}^{2} + 2F_{12} \sigma_{1} \sigma_{2} + F_{1} \sigma_{1} + F_{2} \sigma_{2} = 1$$

3 $$\begin{aligned} F_{11} & = \frac{1}{{X_{\text{T}} X_{\text{C}} }},\quad F_{22} = \frac{1}{{Y_{\text{T}} Y_{\text{C}} }},\quad F_{66} = \frac{1}{{S^{2} }} \\ F_{1} & = \frac{1}{{X_{\text{T}} }} - \frac{1}{{X_{\text{C}} }},\quad F_{2} = \frac{1}{{Y_{\text{T}} }} - \frac{1}{{Y_{\text{C}} }} \\ F_{12} & = - \frac{1}{{2\sqrt {X_{\text{T}} X_{\text{C}} Y_{\text{T}} Y_{\text{C}} } }} \\ \end{aligned}$$

Hashin 2D failure criterion (Hashin 1980) consists of four independent criteria, each of which is associated with one failure mode, as indicated in Eq. (4). One additional strength is required: ply transverse (out-of-plane) shear strength $S_{\text{T}}$. All strengths take absolute values, while all stresses keep their signs.

4 $$\begin{aligned} & {\text{Fiber tensile failure }}\left( {\sigma_{1} > 0} \right){:} \\ & \quad \quad \left( {\frac{{\sigma_{1} }}{{X_{\text{T}} }}} \right)^{2} + \left( {\frac{{\sigma_{6} }}{S}} \right)^{2} = 1 \\ & {\text{Fiber compressive failure }}\left( {\sigma_{1} < 0} \right){:} \\ & \quad \quad \left| {\sigma_{1} } \right| = X_{\text{C}} \\ & {\text{Matrix tensile failure }}\left( {\sigma_{2} > 0} \right){:} \\ & \quad \quad \left( {\frac{{\sigma_{2} }}{{Y_{\text{T}} }}} \right)^{2} + \left( {\frac{{\sigma_{6} }}{S}} \right)^{2} = 1 \\ & {\text{Matrix compressive failure }}\left( {\sigma_{2} < 0} \right){:} \\ & \quad \quad \left( {\frac{{\sigma_{2} }}{{2S_{\text{T}} }}} \right)^{2} + \left[ {\left( {\frac{{Y_{\text{C}} }}{{2S_{\text{T}} }}} \right)^{2} - 1} \right]\frac{{\sigma_{2} }}{{Y_{\text{C}} }} + \left( {\frac{{\sigma_{6} }}{S}} \right)^{2} = 1 \\ \end{aligned}$$

Puck’s theory (Puck and Schürmann 1998, 2002; Puck et al. 2002) divides failure of composites into two categories: fiber failure (FF) and inter-fiber failure (IFF). For FF, 2 criteria corresponding to failure under tension and compression are given in Eq. (5), where $\varepsilon_{{1{\text{T}}}}$ and $\varepsilon_{{1{\text{C}}}}$ are ply longitudinal tensile and compressive failure strains, respectively; *ɛ*_1_ and *ɛ*_6_ are ply longitudinal and in-plane shear strains, respectively; $E_{\text{f1}}$ and $\nu_{\text{f12}}$ are fiber longitudinal elastic modulus and longitudinal major Poisson’s ratio, respectively; the factor $m_{{\sigma {\text{f}}}}$ accounts for the stress magnification effect caused by difference in fiber and matrix moduli in transverse direction.

5 $$\begin{aligned} & {\text{Fiber tensile failure }}\left( {\sigma_{1} \ge 0} \right){:} \\ & \quad \quad \frac{1}{{\varepsilon_{{1{\text{T}}}} }}\left( {\varepsilon_{1} + \frac{{\nu_{\text{f12}} }}{{E_{\text{f1}} }}m_{{\sigma {\text{f}}}} \sigma_{2} } \right) = 1 \\ & {\text{Fiber compressive failure }}\left( {\sigma_{1} < 0} \right){:} \\ & \quad \quad \frac{1}{{\varepsilon_{{1{\text{C}}}} }}\left| {\varepsilon_{1} + \frac{{\nu_{\text{f12}} }}{{E_{\text{f1}} }}m_{{\sigma {\text{f}}}} \sigma_{2} } \right| = 1 - \left( {10\varepsilon_{6} } \right)^{2} \\ \end{aligned}$$

For IFF, three failure modes are defined based on loading conditions and resulting orientation of fracture plane, as indicated in Eq. (6), where $p_{ \bot \parallel }^{\left( + \right)}$ and $p_{ \bot \parallel }^{\left( - \right)}$ are inclination parameters; $R_{ \bot \bot }^{\text{A}}$, $p_{ \bot \bot }^{\left( - \right)}$, and $\sigma_{{6{\text{c}}}}$ are given in Eq. (7).

6 $$\begin{aligned} & {\text{Mode A }}\left( {\sigma_{2} \ge 0} \right){:} \\ & \quad \quad \sqrt {\left( {\frac{{\sigma_{6} }}{S}} \right)^{2} + \left( {1 - p_{ \bot \parallel }^{\left( + \right)} \frac{{Y_{\text{T}} }}{S}} \right)^{2} \left( {\frac{{\sigma_{2} }}{{Y_{\text{T}} }}} \right)^{2} } + p_{ \bot \parallel }^{\left( + \right)} \frac{{\sigma_{2} }}{S} = 1 - \frac{{\sigma_{1} }}{{\sigma_{{ 1 {\text{D}}}} }} \\ & {\text{Mode B }}\left( {\sigma_{2} < 0{\text{ and }}0 \le \left| {\frac{{\sigma_{2} }}{{\sigma_{6} }}} \right| \le \frac{{R_{ \bot \bot }^{\text{A}} }}{{\left| {\sigma_{{6{\text{c}}}} } \right|}}} \right){:} \\ & \quad \quad \frac{1}{S}\left( {\sqrt {\sigma_{6}^{2} + \left( {p_{ \bot \parallel }^{\left( - \right)} \sigma_{2} } \right)^{2} } + p_{ \bot \parallel }^{\left( - \right)} \sigma_{2} } \right) = 1 - \frac{{\sigma_{1} }}{{\sigma_{{ 1 {\text{D}}}} }} \\ & {\text{Mode C }}\left( {\sigma_{2} < 0{\text{ and }}0 \le \left| {\frac{{\sigma_{6} }}{{\sigma_{2} }}} \right| \le \frac{{\left| {\sigma_{{6{\text{c}}}} } \right|}}{{R_{ \bot \bot }^{\text{A}} }}} \right){:} \\ & \quad \quad \left[ {\left( {\frac{{\sigma_{6} }}{{2\left( {1 + p_{ \bot \bot }^{\left( - \right)} } \right)S}}} \right)^{2} + \left( {\frac{{\sigma_{2} }}{{Y_{\text{C}} }}} \right)^{2} } \right]\frac{{Y_{\text{C}} }}{{\left( { - \sigma_{2} } \right)}} = 1 - \frac{{\sigma_{1} }}{{\sigma_{{ 1 {\text{D}}}} }} \\ \end{aligned}$$

7 $$\begin{aligned} R_{ \bot \bot }^{\text{A}} & = \frac{S}{{2p_{ \bot \parallel }^{\left( - \right)} }}\left( {\sqrt {1 + 2p_{ \bot \parallel }^{\left( - \right)} \frac{{Y_{\text{C}} }}{S}} - 1} \right) \\ p_{ \bot \bot }^{\left( - \right)} & = p_{ \bot \parallel }^{\left( - \right)} \frac{{R_{ \bot \bot }^{\text{A}} }}{S} \\ \sigma_{{6{\text{c}}}} & = S\sqrt {1 + 2p_{ \bot \bot }^{\left( - \right)} } \\ \end{aligned}$$

## Experiments

### Specimen fabrication

In this study, two types of glass fiber reinforced composite laminates were involved: biaxial laminates [±*θ*]_S_ (BX*θ*), and trixial laminates [0_2_/±*θ*]_S_ (TX*θ*), where *θ* = 45°, 35°, 25°. All laminates were manufactured using dry glass fiber non-crimp fabrics (NCFs) and epoxy resin system through vacuum-assisted resin infusion process. The biaxial and triaxial NCFs were provided by Chomarat, with the sizing of glass fibers being SE1500. The epoxy resin system consisted of EPIKOTE™ Resin MGS^®^ RIMR 135, and EPIKURE™ Curing Agent MGS^®^ RIMH 137, both of which were provided by Momentive Specialty Chemicals Inc.

**Fig. 1:**
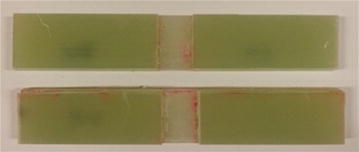
The actual specimens ready for testing

The resin (RIMR 135) and hardener (RIMH 137) were mixed according to a ratio of 100:30 by weight, and the mixture was stirred until two components were mixed thoroughly (no visible clouding inside). The mixture was then placed in a vacuum chamber to degas at a pressure of 0.1 bar for 30 min. The degassed resin/hardener mixture was infused into the vacuum bag until the NCF preforms inside were completely impregnated. After infusion the resin impregnated fabrics were cured in an oven at 60 °C for 18 h. The cured laminates were cut into strips of 145 mm long and 25 mm wide according to ASTM D3410 (2008), using a diamond cutter with water cooling. Since the thickness-to-width ratio of laminates used in this study indicates that they are thin enough, as mentioned in ASTM (2008), the free-edge effect was therefore not taken into account. Tabs made of G10 glass woven fabric/epoxy were attached to those stripes using epoxy adhesive. Surfaces to be in contact with the adhesive were roughened using silicon carbide abrasive paper of CAMI grit size 400 prior to applying the adhesive. The schematic drawing of specimen geometry, as well as actual specimens ready for testing, are shown in Fig. 1.

### Fiber volume fraction measurement

**Fig. 2:**
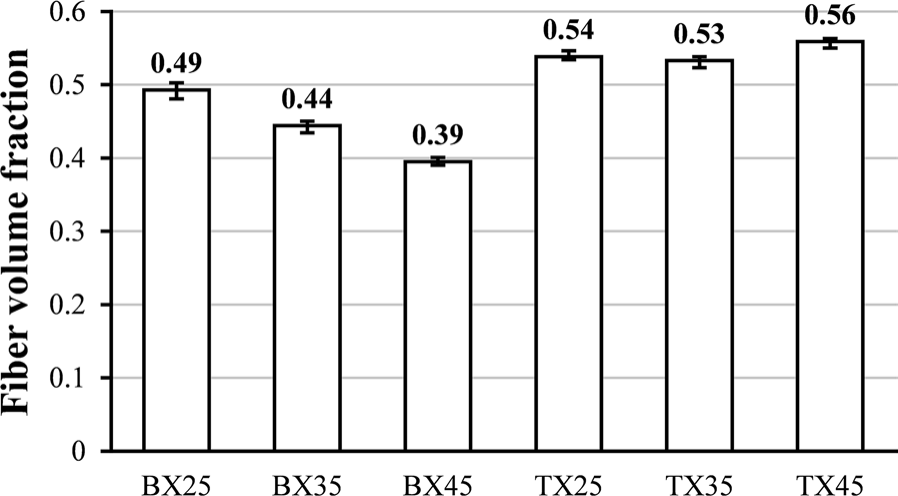
Measured fiber volume fractions of BX and TX laminates

Fiber volume fraction *V*_f_ of BX and TX laminates were computed based on their respective densities measured following water displacement method described in ASTM D792 (1991). As shown in Eq. (8), *V*_f_ can be calculated from densities of fiber, matrix, and composites, denoted as $\rho_{\text{f}}$, $\rho_{\text{m}}$, and $\rho_{\text{c}}$, respectively. The value of $\rho_{\text{f}}$ for glass fiber was determined to be 2.62 and 1.19 g/cm^3^ was used as the value of $\rho_{\text{m}}$ (ASTM 2008). For each laminate, 5 samples with the size of 25 mm × 25 mm were cut from different locations for density measurement. The mean values and coefficients of variation (CV) of fiber volume fractions corresponding to BX25, BX35, BX45, TX25, TX35, TX45 are 0.49 and 1.60, 0.44 and 1.75, 0.39 and 1.16, 0.54 and 1.02, 0.53 and 1.08, 0.56 and 0.90%, respectively, as illustrated in Fig. 2.

8 $$V_{\text{f}} = \frac{{\rho_{\text{c}} - \rho_{\text{m}} }}{{\rho_{\text{f}} - \rho_{\text{m}} }}$$

### Static compression test

**Fig. 3:**
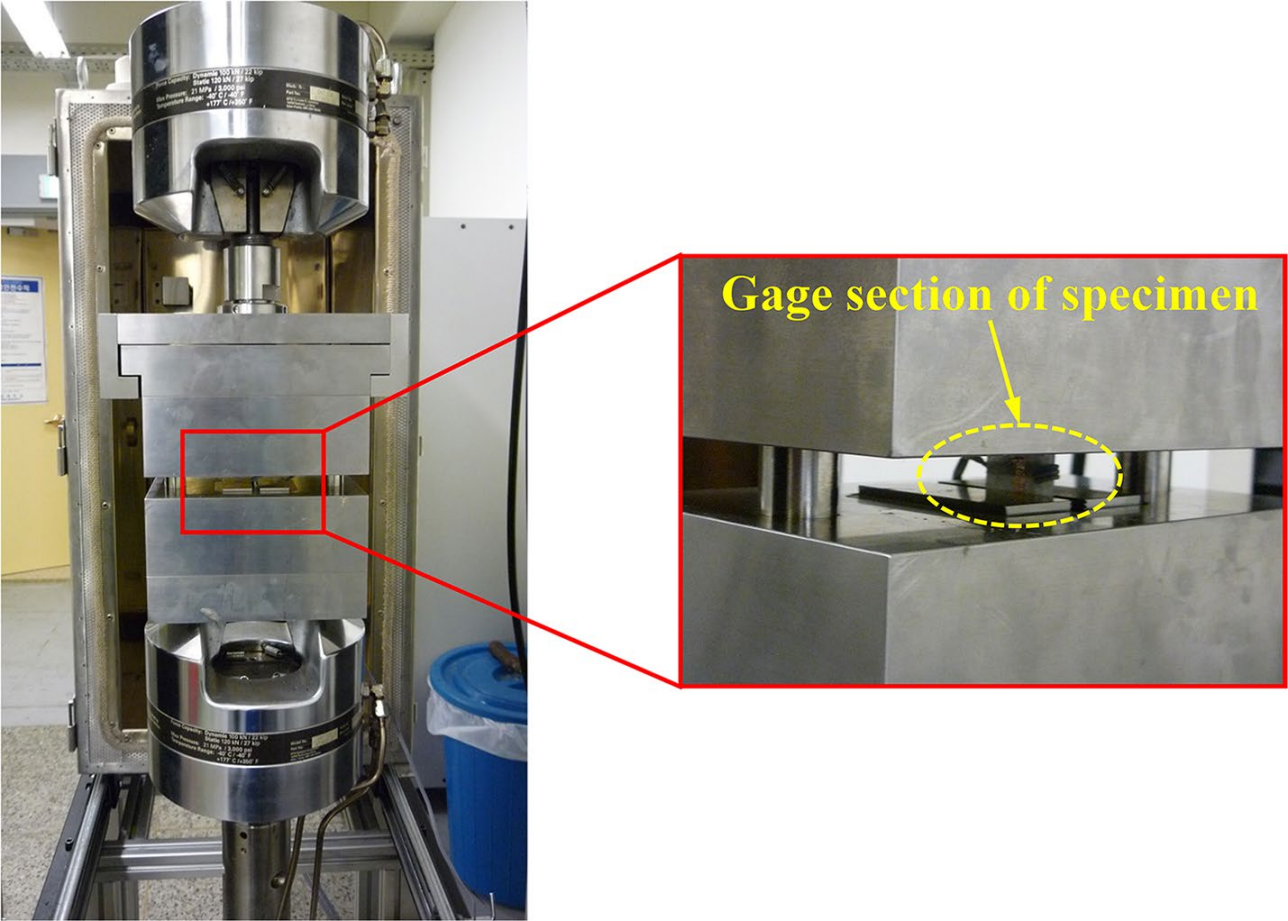
Experimental setup for static compression tests

**Fig. 4:**
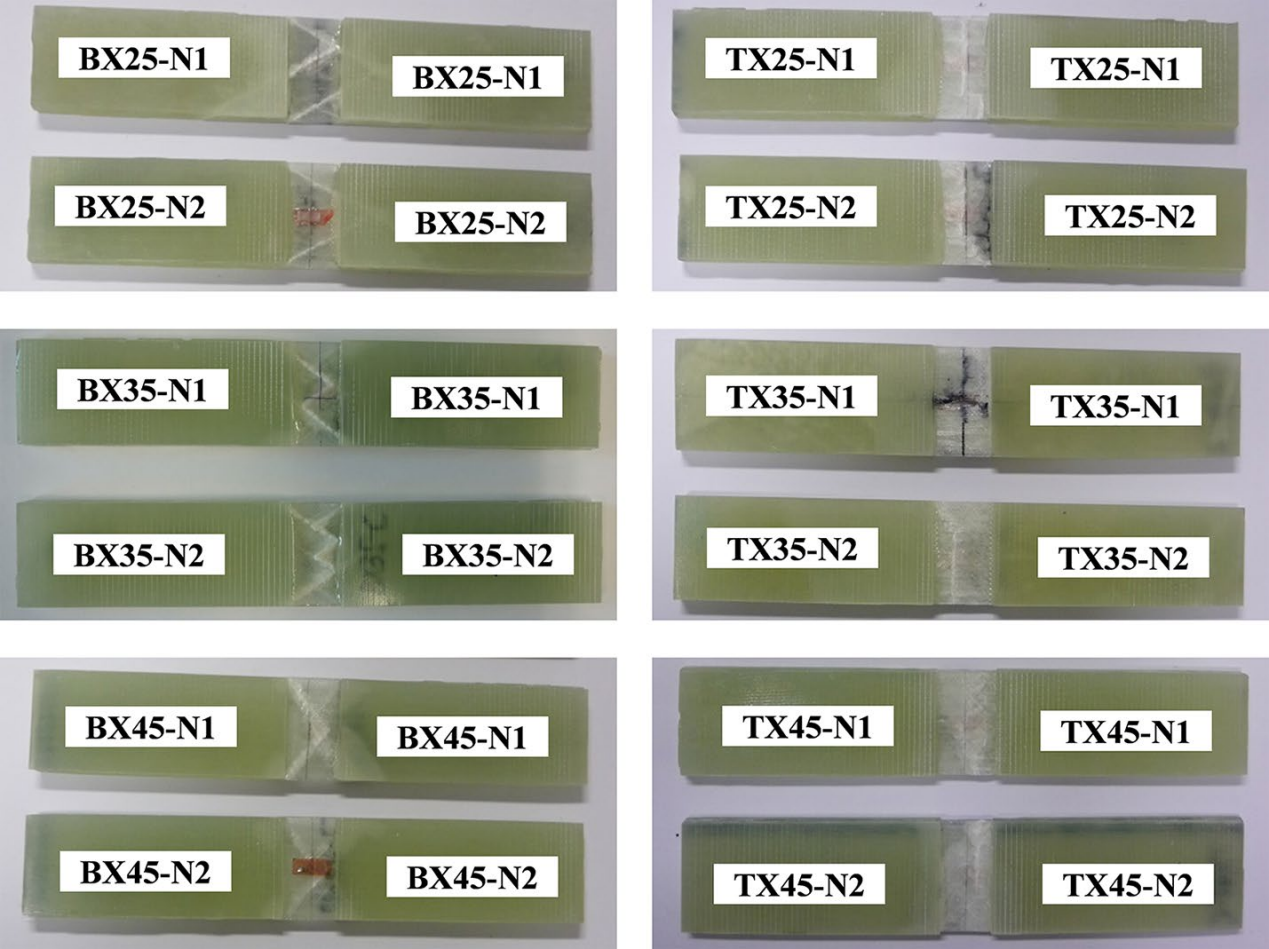
Failed specimens of BX and TX laminates after compression

**Fig. 5:**
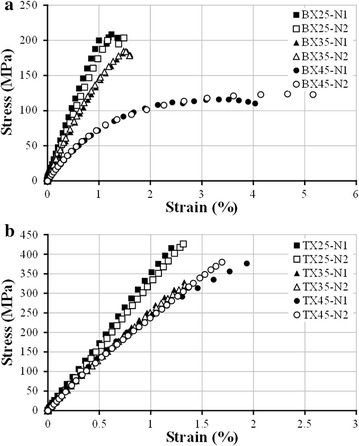
Typical experimentally measured compressive S–S curves of **a** BX*θ*, and **b** TX*θ* laminates (*θ* = 25°, 35°, 45°)

Static compression tests were conducted according to ASTM D3410 (2008) to measure compressive stress–strain (S–S) curves of BX and TX laminates mentioned above. All tests were carried out at room temperature (~25 °C) on an MTS Landmark™ 370.10 servohydraulic test machine, with the crosshead displacement rate of 1 mm/min. For each specimen, a conventional metal foil strain gage was glued to the gage section for strain measurement. The experimental setup is shown in Fig. 3. Failed specimens of BX and TX laminates after compression are displayed in Fig. 4. For all BX laminates, white opaque traces running along fiber directions can be observed through specimen surfaces, which indicates failure of matrix and/or interface in [±*θ*] plies. For TX laminates, those traces are perpendicular to the loading direction, which resulted from fiber fracture in [0] plies. The compressive S–S curves of BX and TX laminates obtained from tests are shown in Fig. 5. The mean values and CVs of uniaxial compressive strengths corresponding to BX25, BX35, BX45, TX25, TX35, TX45 are respectively 208 MPa and 1.73%, 185 MPa and 0.17%, 120 MPa and 3.25%, 421 MPa and 1.14%, 318 MPa and 0.97%, 370 MPa and 1.97%, as indicated in

**Fig. 6:**
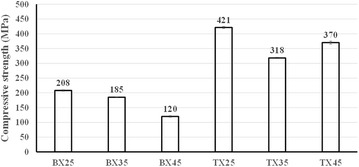
Experimentally measured compressive strengths of BX and TX laminates

Figure 6. It was noticed that the compressive strength of TX35 is lower than that of TX45, which was contrary to expectation.

## Results and discussion

### Parameter determination

The glass fiber and matrix were assumed isotropic. The elastic constants of fiber and matrix were 82 and 3 GPa, respectively; the Poisson’s ratios of fiber and matrix were 0.2 and 0.35, respectively. The ply elastic constants corresponding to BX and TX laminates were computed from Eq. (1), and are tabulated in Table 2. Those values were used in classic laminate theory (CLT) to compute in-plane stresses on each ply.

To estimate ply strengths, strengths of UD glass/epoxy composites with different $V_{\text{f}}$ were collected from various sources (Momentive Specialty Chemicals Inc. 2006; Rolfes et al. 2008; Wichmann 2002; Owens Corning Composite Materials 2008, 2011; Peters et al. 2009; Kaddour and Hinton 2012) and listed in Table 1, where $V_{\text{f}} = 0$ corresponds to pure matrix, while $V_{\text{f}} = 1$ represents fiber. In order to demonstrate that the trend of test data can be described by a regression line, the correlation coefficients *r* of each strength are listed in the last row of Table 1. Note that all *r* values are close to either 1 or −1, implying that liner regression is applicable. The fiber longitudinal compressive strength $X_{\text{C}}$ was not directly taken from the reference; instead, it was estimated using $X_{\text{T}}$ from (Owens Corning Composite Materials 2008) and the ratio between $X_{\text{T}}$ and $X_{\text{C}}$ for E-glass fiber from (Kaddour and Hinton 2012). Therefore, according to the test data shown in Table 1, linear regression analysis was performed to predict the ply strengths for BX and TX laminates, which are listed in Table 2, together with the predicted elastic constants.

In addition to those parameters mentioned above, extra parameters are required in Hashin and Puck failure criteria. For Hashin failure criterion, the ply transverse shear strength $S_{\text{T}}$ was assumed to be 70 MPa for all BX and TX laminates. For FF in Puck failure criterion, $\varepsilon_{{1{\text{T}}}}$ and $\varepsilon_{{1{\text{C}}}}$ were computed by dividing $X_{\text{T}}$ and $X_{\text{C}}$ respectively by $E_{{{\text{f}}1}}$. The values of $E_{\text{f1}}$ and $\nu_{\text{f12}}$ were 82 GPa and 0.2, respectively. The value of $m_{{\sigma {\text{f}}}}$ was 1.3 (Puck and Schürmann 1998). For IFF in Puck failure criterion, the values of $p_{ \bot \parallel }^{\left( + \right)}$ and $p_{ \bot \parallel }^{\left( - \right)}$ were 0.3 and 0.25, respectively (Puck and Mannigel 2007).

### Strength prediction versus test data

With parameters determined in the above section, Tsai–Wu, Hashin, and Puck failure criteria were employed to predict uniaxial compressive strengths of BX and TX laminates. In all analyses linear elasticity was assumed, and thermal residual stress was neglected. The average stress undertaken by the laminate was treated as laminate strength when any ply failure occurred, i.e. first-ply-failure (FPF) scheme.

**Fig. 7:**
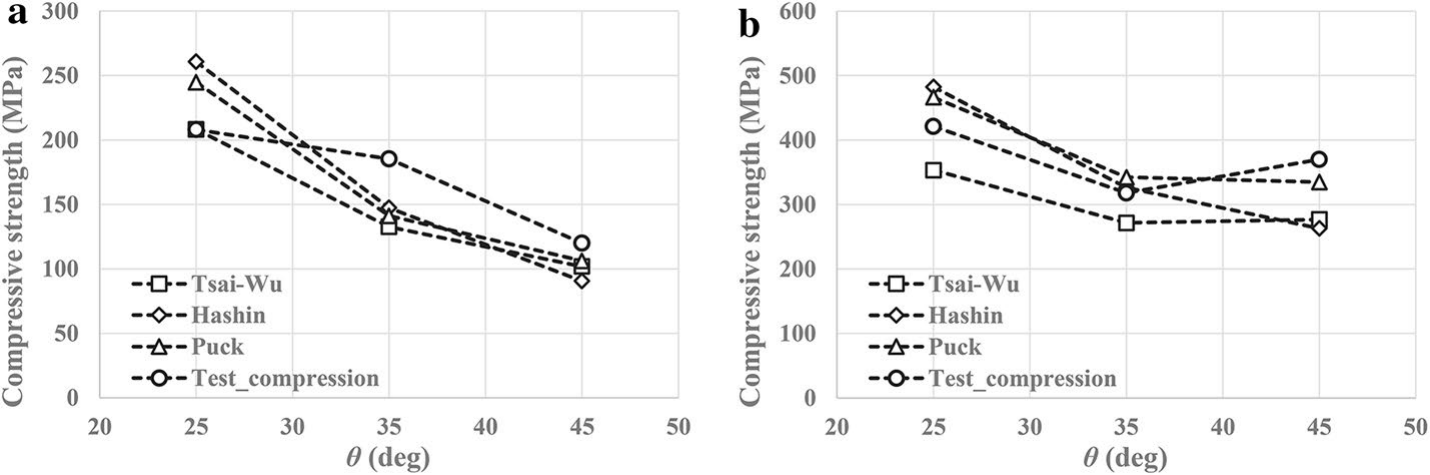
Comparison between prediction and test data of compressive strengths of **a** BX laminates, and **b** TX laminates

 Figure 7 shows comparison between predictions from three failure criteria and test data of compressive strengths of BX and TX laminates. For BX25 and BX35, due to the existence of on-axis transverse tensile stress in angled plies, the failure mode predicted by Hashin criterion was matrix tensile failure, while Puck predicted Mode A IFF. For BX45 however, Hashin predicted matrix compressive failure, and Puck predicted Mode B IFF, owing to on-axis transverse compressive stress in angled plies. It can be seen from Fig. 7a that Tsai–Wu criterion gave the best prediction for BX25, and at the same time predictions from Hashin and Puck were ~25% higher than the prediction from Tsai–Wu. For BX35 however, although predictions yielded from all three criteria were similar, they were ~25% lower than experimental results. For BX45, all three criteria gave similar predictions, which are slightly lower than test data. The similarities in predictions from Hashin and Puck criteria were expected, due to the commonality in principles employed by the two criteria. For BX25 and BX35, the magnitude of longitudinal compressive and transverse tensile stress decreases as ply angle increases. Therefore, the contribution of those stresses to failure in Tsai–Wu criterion does not increase significantly as ply angle increases, leading to the reduction in the difference between predictions from Tsai–Wu and the other two criteria. In general, all criteria are able to predict the trend of reduction in compressive strength with increasing ply angle in BX laminates, i.e. the strength decreases as ply angle increases.

In the case of TX laminates, Hashin criterion predicted matrix tensile failure of angled plies in TX25, and matrix compressive failure of angled plies in TX35 and TX45. Puck criterion predicted Mode A IFF of angled plies in TX25, and Mode B IFF of angled plies in TX35 and TX45. Only Tsai–Wu gave 0° ply failure for all three types of TX laminates. Hashin and Puck give similar overestimation of TX25 compressive strength, while Tsai–Wu underestimates the value. For TX35, predictions generated by Hashin and Puck show good agreement with test data, whereas Tsai–Wu still gives a value smaller than test data. For TX45, Tsai–Wu and Hashin have similar predictions, which are ~25% lower than test result, but prediction from Puck criterion is much closer to test result. Despite the fact that Hashin and Puck criteria give fair predictions at certain ply angles, only Tsai–Wu criterion captures the unexpected variation of compressive strength of TX laminates with ply angle.

As explained previously, FPF was used in all analyses performed in this study, which is reasonable for BX laminates since all plies are in the same stress state. Nevertheless, FPF might lead to underestimation of actual laminate strength if remaining plies still have enough load-bearing capability. For TX25, TX35, and TX45, both Hashin and Puck predicted the occurrence of FPF in angled plies, whereas Tsai–Wu predicted FPF in 0° plies for all three layups. According to CLT, 0° plies are major load-bearing elements in TX laminates, so the fracture of 0° plies would cause catastrophic impairment to the load-bearing capability of the entire laminate, as demonstrated by the specimens of TX laminates after compressive failure shown in Fig. 4. Therefore, even though the predictions of TX compressive strengths given by Tsai–Wu criterion are less accurate at certain specific ply angles when comparing with Hashin and Puck criteria, they do follow the same tendency of strength variation with ply angle, as exhibited by test data.

**Fig. 8:**
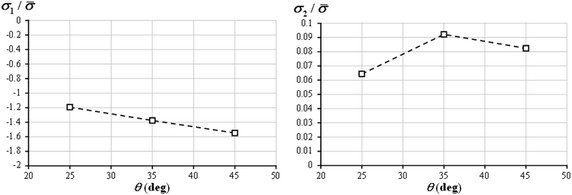
Longitudinal and transverse stresses in 0° plies of TX laminates normalized by laminate stress

**Fig. 9:**
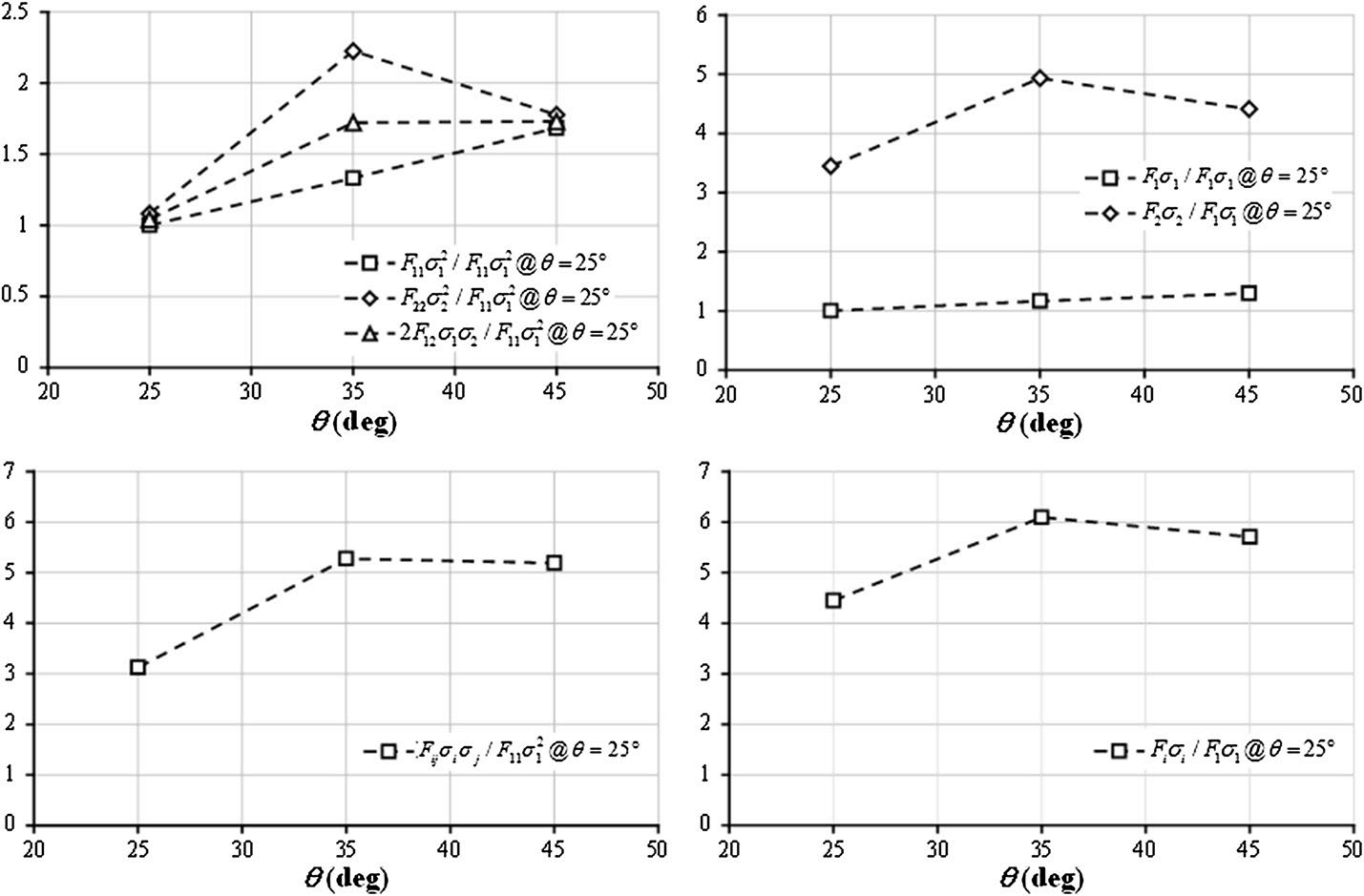
Contribution of each term in Tsai–Wu criterion to failure determination, where quadratic terms are normalized by $F_{11} \sigma_{1}^{2}$ at *θ* = 25°, and linear terms are normalized by $F_{1} \sigma_{1}$ at *θ* = 25°

Since Tsai–Wu failure criterion captured the variation of TX compressive strengths with ply angle, endeavors were made to explain the cause of the experimentally observed lower TX35 compressive strength. As shown in Fig. 8, the normalized longitudinal compressive stress in 0° plies increases as *θ* increases; while the normalized transverse tensile stress in 0° plies attains its maximum at *θ* = 35°. Unlike Hashin and Puck criteria, Tsai–Wu criterion does not distinguish any failure modes. In order to determine the dominant stress component, the contribution of each term in Tsai–Wu criterion to failure determination was plotted in Fig. 9. The quadratic terms were normalized by the value of $F_{11} \sigma_{1}^{2}$ at *θ* = 25°, and linear terms were normalized by the value of $F_{1} \sigma_{1}$ at *θ* = 25°. It is clearly observed that both the quadratic and linear terms associated with transverse stress reach their maxima at *θ* = 35°. Additionally, the sum of all quadratic terms and the sum of all linear terms also reach their respective peaks at *θ* = 35°. Especially, considering the magnitude, the ply transverse stress is the dominant factor in failure determination. Consequently, it can be concluded that the transverse tensile stress in 0° plies is the major cause of lower compressive strength of TX35 laminate. When axial compressive load is applied, the influence of the change in longitudinal compressive stress on transverse strain is smaller when the ply angle is between 25° to 35° than the case when the ply angle is between 35° to 45°; meanwhile, the influence of the change in longitudinal compressive stress on longitudinal strain remains the same. As a result, the transverse stress, which is mainly determined by transverse strain in this case, reaches the maximum at *θ* = 35°. Understanding the cause of such an unexpected phenomenon is of practical importance, since the shallow-angled BX and TX laminates might replace their conventional counterparts with ±45° angled plies in structures, and bring extra benefits. For example, as mentioned by Hayat and Ha (2015), Ha et al. (2014), the application of shallow-angled skins in wind turbine blades can improve the bending stiffness and strength, while reducing the thickness of spar cap and the overall blade mass.

## Conclusion

In this paper, BX and TX laminates with shallow angles (*θ* = 45°, 35°, 25°) were fabricated using glass fiber NCFs and epoxy resin through vacuum-assisted resin infusion process. The average fiber volume fraction of cured BX25, BX35, and BX45 laminates was respectively measured as 0.49, 0.44, and 0.39; those values for TX25, TX35, and TX45 were 0.54, 0.53, and 0.56, respectively. Static compression tests were carried out following ASTM D3410, using specimens manufactured from aforementioned BX and TX laminates. The average uniaxial compressive strengths of BX25, BX35, BX45, TX25, TX35, and TX45 were measured as 208, 185, 120, 421, 318, and 370 MPa, respectively. The stress–strain curves show that, for BX laminates, both elastic moduli and compressive strengths decrease as the ply angle *θ* increases; for TX laminates however, although the variation of elastic moduli with *θ* follows the same trend as in the case of BX laminates, the lowest compressive strength is achieved by BX35 instead of BX45.

To predict the experimentally measured compressive strengths of BX and TX laminates, Mori–Tanaka type mean field homogenization method was utilized to estimate elastic constants of plies with different $V_{\text{f}}$ first. Ply strengths corresponding to different $V_{\text{f}}$ were then estimated using linear regression of a collection of test data. Finally Tsai–Wu, Hashin, and Puck failure criteria were employed to predict compressive strengths of BX and TX laminates. It turned out that, for BX laminates, all three criteria gave similar predictions, and the predicted compressive strength decreases with increasing ply angle, which conforms to experimental observation. For TX laminates, although Hashin and Puck outperformed Tsai–Wu in terms of accuracy at certain ply angles, only Tsai–Wu was able to predict the unexpected strength variation of TX laminates with ply angle. Further analysis revealed that the transverse tensile stress in 0° plies of TX laminates is the dominant factor in failure determination of Tsai–Wu criterion, and the stress attains its maximum when *θ* = 35°.

## Abbreviations

PMC: polymeric matrix composite
BX: biaxial laminate
TX: triaxial laminate
UD: unidirectional composite
FF: fiber failure
IFF: inter-fiber failure
WWFE-I: the first world-wide failure exercise
NCF: non-crimp fabric
CV: coefficient of variation
S–S: stress–strain
CLT: classic laminate theory
FPF: first-ply-failure
